# Interleukin-6, vascular endothelial growth factor and transforming growth factor beta 1 in canine steroid responsive meningitis-arteritis

**DOI:** 10.1186/1746-6148-9-23

**Published:** 2013-02-04

**Authors:** Arianna Maiolini, Meike Otten, Marion Hewicker-Trautwein, Regina Carlson, Andrea Tipold

**Affiliations:** 1 Department of Small Animal Medicine and Surgery, University of Veterinary Medicine, Hannover, Germany; 2 Center for Systems Neuroscience, Hannover, Germany; 3 Veterinary Practice Peter Gravert and Dr. Volker Otten, Gettorf, Germany; 4 Department of Pathology, University of Veterinary Medicine, Hannover, Germany

**Keywords:** Steroid responsive meningitis-arteritis (SRMA), Interleukin-6 (IL-6), Transforming growth factor beta 1 (TGF-β_1_), Vascular endothelial growth factor (VEGF), Cerebrospinal fluid (CSF), Dog, Central nervous system (CNS)

## Abstract

**Background:**

Steroid Responsive Meningitis-Arteritis (SRMA) is a common cause of inflammation of the canine central nervous system (CNS). To investigate if transforming growth factor beta 1 (TGF-β_1_), interleukin-6 (IL-6) and vascular endothelial growth factor (VEGF) are involved in the production of excessive immunoglobulin A (IgA), the induction of acute phase proteins and in the development of a systemic necrotizing vasculitis, characteristic of SRMA, these three signalling proteins were evaluated.

**Results:**

Cerebrospinal fluid (CSF) and serum samples of dogs during the acute phase of SRMA (SRMA) were tested for IL-6, VEGF and TGF- β_1_. Results were compared to those of dogs affected with SRMA during treatment (SRMA Th) and during relapse (SRMA R), to dogs with other meningoencephalomyelitides (ME), with miscellaneous non-inflammatory diseases of the CNS (CNS-Mix), with idiopathic epilepsy (IE), with systemic inflammatory diseases (Syst. Infl.) and with healthy dogs (Healthy). Concentrations of IL-6 and VEGF in CSF were significantly elevated in the SRMA group compared to the other disease categories (p < 0.05). The CSF concentrations of TGF-β_1_ were increased in SRMA group, but statistically significant differences were found only in comparison with Healthy and CNS-Mix groups. No differences were detected in the serum concentrations of TGF-β_1_ between the different groups. In untreated SRMA patients, a positive correlation (r_Spear_ = 0.3549; *P* = 0.0337) between concentrations of TGF-β_1_ and IgA concentration was found in CSF, while concentrations of IL-6 and VEGF in CSF positively correlated with the degree of pleocytosis (r_Spear_ = 0.8323; *P* < 0.0001 and r_Spear_ = 0.5711; *P* = 0.0166, respectively).

**Conclusions:**

Our results suggest that these three signalling proteins are biomarkers of disease activity in SRMA. VEGF might play an important role in the development of a systemic arteritis. TGF-β_1_ is considered to be involved in the excessive IgA production, while IL-6 in the pleocytosis. The combined intrathecal increase of TGF-β_1_ and IL-6 detected in SRMA could possibly force CD4 progenitors to differentiate towards the newly described Th17 lymphocyte subset and enhance the autoimmune response.

## Background

Steroid Responsive Meningitis-Arteritis (SRMA) is a relatively common inflammatory disease of the canine central nervous system (CNS) [1], being the primary cause of meningitis [2,3] and one of the most common causes of fever in referred dogs [4]. Typically affected dogs manifest severe neck pain in addition to signs of systemic illness, such as fever and lethargy. The meningeal and systemic inflammation is usually reflected by laboratory findings, such as pleocytosis and leukocytosis [5] and the elevation of acute phase proteins [6-8]. A concurrent increased intrathecal and systemic production of immunoglobulin A (IgA) has been found in dogs affected with SRMA [9], and its determination supports the diagnosis [10]. Recent studies underlined the importance of a Th2-mediated immune response in SRMA patients [11,12], but the exact etiopathogenesis of the disease is still unclear.

Signalling proteins are molecules, such as cytokines, growth factors, hormones and neurotransmitters, that interact with receptors. Vascular Endothelial Growth Factor (VEGF) is a potent, multifunctional factor, which regulates angiogenesis and blood vessel permeability [13,14]. In the last two decades the role of VEGF has been widely investigated in systemic vasculitides [15-17] and malignant tumours [18,19]. In veterinary medicine VEGF has been studied in some inflammatory diseases [20] and tumours [21,22], but its role in systemic vasculitis is still unknown. Interleukin 6 (IL-6) is a proinflammatory cytokine, which activates lymphocytes, increases antibody production, induces fever and acute-phase protein production [23], while Transforming Growth Factor Beta 1 (TGF-β_1_) induces a class switching of B lymphocytes towards IgA production [24].

To test the hypothesis that the signalling proteins VEGF, IL-6 and TGF-β_1_ are involved in the pathogenesis of SRMA, these proteins were determined in cerebrospinal fluid (CSF) and serum. We hypothesize that enhanced VEGF production is responsible for the increased vascular permeability (vasculitis) observed in SRMA dogs, while IL-6 and TGF-β_1_ are responsible for the excessive IgA production and the systemic inflammatory response, especially for acute phase protein production and development of T-helper cell subtypes.

## Methods

### Serum and cerebrospinal fluid (CSF) samples

Serum and cerebrospinal fluid (CSF) samples were collected from client-owned dogs, presented to the Neurology Service of the Department of Small Animal Medicine and Surgery, University of Veterinary Medicine, Hannover, Germany, according to the Universities rules.

All dogs underwent a standard neurological examination performed either by a veterinary neurology resident or a board-certified veterinary neurologist. Depending on the diagnosis, animals were assigned to one of the following groups (see Table 1): ‘SRMA untreated’ (SRMA), ‘SRMA relapse’ (SRMA R), ‘SRMA therapy’ (SRMA Th), ‘idiopathic epilepsy’ (IE), ‘Meningoencephalomyelitides’ (ME), and ‘miscellaneous non-inflammatory CNS diseases’ (CNS-Mix).

**Table 1:** Distribution of disease categories

**Groups**	**Findings and number of dogs**
**SRMA untreated (SRMA)**	Dogs with fever, cervical pain, neutrophilic leukocytosis and pleocytosis. These dogs were not pre-treated with glucocorticosteroid (n = 36)
**SRMA relapse (SRMA R)**	Dogs with a previous diagnosis of SRMA in which clinical signs recurred despite glucocorticosteroid treatment (n = 9)
**SRMA therapy (SRMA Th)**	Dogs with previous diagnosis of SRMA under long-term glucocorticosteroid treatment. These dogs did not show any clinical signs at the time of sampling (n = 20)
**Idiopathic epilepsy (IE)**	Dogs with clinical, CSF, MRI findings consistent with idiopathic epilepsy (n = 22)
**Meningoencephalomyelitides (ME)**	Dogs with clinical, CSF, MRI and/or pathological findings consistent with meningoencephalomyelitides: granulomatous meningoencephalomyelitis (n = 14), necrotising encephalitides (n = 1), meningoencephalomyelitis of unknown origin (n = 8), infectious encephalitides (n = 24)
**Miscellaneous non-inflammatory CNS diseases (CNS-Mix).**	Dogs with miscellaneous not primarily inflammatory neurological diseases: intervertebral disc disease (n = 9), neoplasia of the CNS (n = 24), cerebrovascular disease (n = 4), degenerative myelopathy (n = 1)
**Systemic inflammatory diseases (Syst. Inflam.)**	Dogs affected with systemic inflammatory diseases, but not suffering from neurological conditions (n = 16)
**Healthy**	Healthy dogs (n = 20)

CSF: cerebrospinal fluid; CNS: central nervous system; MRI: magnetic resonance imaging; SRMA: steroid-responsive meningitis-arteritis.

Diagnoses were based on the results of general and neurological examinations, complete blood cell count (CBC), blood chemistry, CSF analysis and further specific diagnostic procedures if considered useful to achieve a definitive or ‘highly likely’ diagnosis (e.g. radiographic and ultrasound examination, electrodiagnostics, computed tomography, magnetic resonance imaging, surgery, histopathology).

Dogs not suffering from neurological conditions, but otherwise affected with systemic inflammatory diseases were also included in the study (‘Syst. Inflam.’).

Healthy dogs with a normal general and neurological examination and laboratory values in the reference range served as negative control group (‘Healthy’).

CSF and serum samples were stored at −20°C until batch analyzed.

The study was conducted following the ethical guidelines of the University for Veterinary Medicine Hannover. The experimental protocols and procedures in healthy dogs were performed in accordance with the European Communities Council Directive of 24 November 1986 (86/609/EEC) and were approved by the authorities in Lower Saxony (animal experiment number 33.42502/05-12.05).

### Quantification of IL-6

Serum and CSF IL-6 concentrations were measured with a commercially available canine-specific Enzyme Linked Immunosorbent Assay (ELISA) following manufacturer’s instructions (Quantikine Canine IL-6 Immunoassay; R&D Systems, Minneapolis, MN, USA). The mean minimum detectable value given by the manufacturer was 6.1 pg/mL. Values lower than 6.1 pg/mL, were considered negative for IL-6. If the value ranged between 6.1 pg/mL and the highest dilution of the standard curves (31.25 pg/mL) a fixed value of 3 pg/mL was assigned for statistical analysis. If the value exceeded the highest value of the standard curve (2000 pg/mL), the sample was diluted and measured again.

All samples were analyzed in duplicates and mean values were calculated.

The IL-6 bioactivity in serum and CSF was verified testing its ability to stimulate proliferation of B9 cell line, a mouse B cell murine hybridoma cell line that requires IL-6 for survival and proliferation (DSMZ No ACC 211, German Collection of Microorganisms and Cell Cultures, DSMZ, Braunschweig, Germany) [25-28].

Cells were cultured at 37°C and 5% CO_2_ in Rosewell Park Memorial Institute (RPMI) 1640 medium with L-glutamine (Gibco® RPMI Media 1640) containing 10% heat-inactivated fetal bovine serum, 50 pg/mL recombinant canine IL-6 (rcIL-6) (Recombinant Canine IL-6; R&D Systems, Minneapolis, MN, USA) and 50 μM 2-mercaptoethanol.

Cells for the assay were washed twice in the above described medium without IL-6. For each assay, a standard curve was prepared using rcIL-6 with serial threefold dilutions starting at 200 pg/mL.

Samples were tested in duplicates at dilutions ranging from 1:2 to 1: 240.

For the IL-6 bioactivity assay B9 cells (5000 cells/well) were incubated for 48 hours with rcIL-6 standard dilutions or diluted canine CSF and serum samples in opaque 96-well microtiter plates (F96 MicroWell™ Plates, Nunc™, Denmark). Cell proliferation and viability was quantified using the CellTiter-Glo® Luminescent Cell Viability Assay (Promega, Madison, WI), according to manufacturer’s recommendations. Luminescence in each well was measured 30 min after reagent addition using a scanning multiwell spectrophotometer equipped with the analysis software Gen 5 (Synergy2 HT multi-mode microplate reader, BioTek Instruments Inc., Bad Friedrichshall Germany).

### Quantification of VEGF

Serum and CSF VEGF concentrations were measured with commercially available canine-specific ELISA following manufacturer’s instructions (Quantikine Canine VEGF Immunoassay, R&D Systems, Minneapolis, MN, USA). The minimum detectable values given by the manufacturer were < 9.8 pg/mL for CSF and < 19.5 pg/mL for serum. Similarly as described for the IL-6 ELISA, a content of 0 pg/mL was assigned if the amount of VEGF detected was less than the minimum detectable concentration.

If the amount ranged between 9.8 pg/mL for CSF or 19.5 pg/mL for serum and the highest dilution of the standard curves (19.5 pg/mL and 39.1 pg/mL, respectively) a fixed value of 2 pg/mL and 3.9 pg/mL instead of zero was assigned for statistical analysis.

All samples were analyzed in duplicates and mean values were calculated.

### Quantification of TGF-β_1_

Serum and CSF TGF-β_1_ concentrations were measured with a commercially available ELISA following manufacturer’s instructions (Human TGF-beta 1 DuoSet, R&D Systems, Minneapolis, MN, USA ). This assay is designed to detect the biologically active natural and recombinant human TGF-β_1._ Canine TGF-β_1_ shares 94% nucleotide sequence identity to human TGF-β_1_[29]. Therefore, we suspected a relative high cross-reactivity that allows the measurement of canine TGF-β_1_ by using this human ELISA Development Kit. The final dilution was 1:15 for the CSF samples and 1:30 and 1:60 for the serum samples. All dilutions were tested in duplicates on 96-well microplates (Nunc-Immuno™-Plate, Nunc™, Denmark). The absorbance was recorded at a wavelength of 450 nm using a plate reader (Dynatech, Denkendorf, Germany).

Antibodies were tested by immunohistochemistry (IHC) detecting canine TGF-β_1_ on canine tissue. Different canine tissues (activated lymph nodes, brain tumour, brain and spinal cord from SRMA affected dogs) were stained with the capture antibody of the previously mentioned ELISA-Kit. All tissues were routinely formalin-fixed and paraffin-embedded and the sections were mounted on positively charged slides (SuperFrost Plus®, Menzel-Gläser, Wiesbaden, Germany). Tissue sections were deparaffinised and the endogenous peroxidase was blocked with 0.5% H_2_O_2_ in 70% ethanol over 30 minutes. After washing the slides with PBS three times for five minutes, citrate buffer (10 minutes, autoclave, 121°C) was used for antigen retrieval. The capture antibody of the ELISA-Kit, a monoclonal mouse-anti-human TGF-β_1_-antibody was diluted 1:40 in PBS (pH 7.5) with 1% bovine serum albumin (BSA) and incubated overnight by 4°C. As secondary antibody we used a biotinylated goat-anti-mouse-antibody (Vector Laboratories, Burlingame, USA) (diluted 1:200 in PBS, incubation for 45 minutes, room temperature). Staining was completed using an ABC Kit (Vector Laboratories, Burlingame, USA).

### Quantification of IgA

Serum and CSF IgA concentration were measured in 31 dogs with ‘untreated SRMA’ using an ELISA, as previously described [9].

### Data analysis

Data were analyzed for normal distribution using the Kolmogorov-Smirnov test, and non-parametric tests were used to analyze data not conforming to a Gaussian distribution.

In addition to descriptive methods, the Kruskal-Wallis test was used to evaluate the analysis of variance. Multiple comparisons between the SRMA groups and groups of other diseases were performed using the Wilcoxon rank-sum test.

The Spearman’s rank correlation coefficients (r_Spear_) were calculated in the “SRMA untreated” group to detect correlations between signalling proteins and different parameters, such as IgA concentrations, complete blood cell count and CSF cell count.

Data were analyzed by using the statistical software package (GraphPad Prism®, version 5; GraphPad Software, La Jolla, CA, USA) and values of P < 0.05 were considered significant.

## Results

CSF and serum samples of 172 dogs were collected and analysed. In additional 36 dogs only serum was collected. The small amount of some of the samples did not allow performing the quantification of all three signalling proteins in each sample. The distribution of the samples for each ELISA is shown in Table 2.

**Table 2:** Number of CSF and serum samples analysed for each signalling protein in each group

**Groups**	**IL-6**		**VEGF**		**TGF-ß1**	
**n of samples**	**CSF**	**Serum**	**CSF**	**Serum**	**CSF**	**Serum**
**SRMA**	26	28	21	28	36	35
**SRMA R**	9	8	9	8	0	0
**SRMA Th**	41	44	41	45	0	0
**ME**	13	13	11	17	39	35
**CNS-Mix**	21	22	18	20	29	26
**Syst. Inflam.**	7	12	3	16	0	0
**IE**	22	16	14	14	0	0
**Healthy**	8	8	8	8	20	15

IL-6: interleukin-6; VEGF: vascular endothelial grow factor; TGF-β_1_: Transforming growth factor beta; CSF: cerebrospinal fluid; CNS: central nervous system; SRMA: steroid-responsive meningitis-arteritis; Syst Inflam: systemic inflammatory diseases; ME: other inflammatory CNS diseases; CNS-Mix: miscellaneous non-inflammatory CNS diseases; IE: idiopathic epilepsy.

### Quantification of IL-6

**Figure 1:**
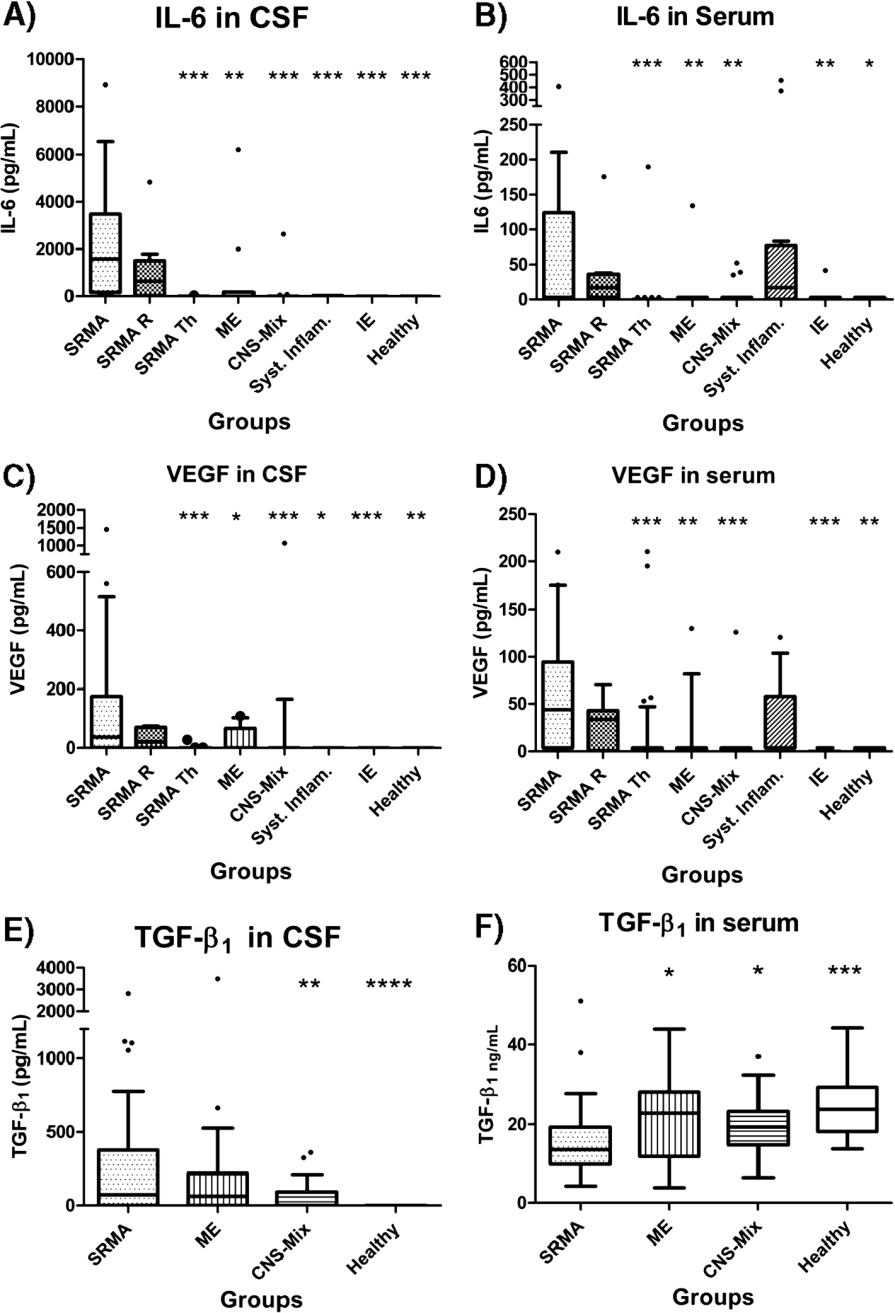
**Quantification of signalling proteins.** Quantification of signalling proteins: IL-6 in CSF (**A**) and serum (**B**), VEGF in CSF (**C**) and serum (**D**), TGF-β_1_ in CSF (**E**) and serum (**F**). Boxes contain values from 1st to 3rd quartile, lines inside boxes indicate median values, endpoints of vertical lines display 5th–95^th^ percentile and · represent the outliers. Asterisks indicate statistically significant differences from the disease category ‘SRMA untreated’ (* *P* < 0.05; ** *P* < 0.01; *** *P* < 0.005). IL-6: interleukin-6; VEGF: vascular endothelial grow factor; TGF-β_1_: Transforming growth factor beta 1. CSF cerebrospinal fluid; CNS: central nervous system; SRMA: steroid-responsive meningitis-arteritis; ME: other inflammatory CNS diseases; CNS-Mix: miscellaneous non-inflammatory CNS diseases; Syst Inflam: systemic inflammatory diseases; IE: idiopathic epilepsy.

IL-6 concentrations in CSF and serum and results of the statistical analysis are illustrated in Figure 1A and 1B and Table 3. The highest concentrations of IL-6 were detected in CSF of dogs with SRMA (median 1582 pg/mL; range 163.1-3473 pg/mL). This group differed significantly from the other disease categories, with the exception of dogs with relapsed SRMA (median 637.7 pg/mL; range 17.19-1507 pg/mL).

**Table 3:** Concentration of signalling proteins in different disease categories

		**SRMA**	**SRMA R**	**SRMA Th**	**Syst Inflam**	**ME**	**CNS-Mix**	**IE**	**Healthy**
**IL-6**	**CSF**	1582	637.7	0	0	3	0	0	3
	(pg/mL)	(163.1-3473)	(17.19-1507)	(0–1.5)	(0–31.25)	(0–175.6)	(0–3)	(0–3)	(0.75-3)
	**Serum**	3	17.13	0	17.13	0	0	1.5	3
	(pg/mL)	(3–124)	(3–36.24)	(0–0)	(0–77.1)	(0–3)	(0–3)	(0–3)	(0–3)
**VEGF**	**CSF**	36.31	20.80	0	0	0	0	0	0
	(pg/mL)	(1–175.2)	(0–69.96)	(0–0)	(0–0)	(0–65.71)	(0–0)	(0–0)	(0–0)
	**Serum**	43.92	33.71	3.9	3.9	0	0	0	3.9
	(pg/mL)	(3.9-94.5)	(0.98-42.9)	(0–3.9)	(3.9-57.8)	(0–3.9)	(0–3.9)	(0–0)	(1.95-3.9)
**TGF-β**_**1**_	**CSF**	90	n.a.	n.a.	n.a.	58	0	n.a.	0
	(pg/mL)	(0–429)				(0–214)	(0–95)		(0–0)
	**Serum**	13.57	n.a.	n.a.	n.a.	22.84	19.31	n.a.	23.84
	(ng/mL)	(9.93-19.27)				(11.86-27.96)	(14.77-23.25)		(18.19-29.16)

IL-6: interleukin-6; VEGF: vascular endothelial grow factor; TGF-β_1_: Transforming growth factor beta; CSF: cerebrospinal fluid; CNS: central nervous system; SRMA: steroid-responsive meningitis-arteritis; Syst Inflam: systemic inflammatory diseases; ME: other inflammatory CNS diseases; CNS-Mix: miscellaneous non-inflammatory CNS diseases; IE: idiopathic epilepsy; n.a.: not available. Data are given as median and range (25th–75th percentile).

Similarly, high concentrations of IL-6 were detected in serum in the ‘SRMA’ group (median 3 pg/mL; range 3–124 pg/mL), ‘SRMA R’ group (median 17.13 pg/mL; range 3–36.24 pg/mL) and ‘Syst. Inflam.’ group (median 17.13 pg/mL; range 0–77.1 pg/mL). In the remaining groups the IL-6 concentration was significantly lower than in the ‘SRMA’ group.

Approximately 30% of the samples used for the ELISA were also tested with the bioassay.

The B9 cells could proliferate only when incubated with samples with high IL-6 values measured by the ELISA.

### Quantification of VEGF

VEGF concentrations in CSF and serum and results of the statistical analysis are illustrated in Figure 1C and 1D and Table 3.

The highest concentrations of VEGF were detected in CSF (median 36.31 pg/mL; range 1–175.2 pg/mL) and serum (median 43.92 pg/mL; range 3.9-94.5 pg/mL) of dogs with SRMA. Similarly, high concentrations of VEGF were found in CSF (median 20.80 pg/mL; range 0–69.96 pg/mL) and serum (median 33.71 pg/mL; range 0.98-42.9 pg/mL) of dogs with relapsed SRMA and in serum (median 3.9 pg/mL; range 3.9-57.8 pg/mL) of dogs with a systemic inflammatory disease. In the remaining groups the VEGF concentrations were significantly lower than the ones of the ‘SRMA’ group.

### Quantification of TGF-β_1_

TGF-β_1_ concentrations in CSF and serum and results of the statistical analysis are illustrated in Figure 1E and F and Table 3.

Compared with the ‘Healthy’ group, TGF-β_1_ concentrations in CSF were significantly higher in the ‘SRMA’ (median 90 pg/mL; range 0–429 pg/mL) and the ‘ME’ (median 58 pg/mL; range 0–214 pg/mL) groups. In serum the ‘SRMA’ group showed the lowest concentrations of TGF-β_1_ (median 13.57 ng/mL, range 9.93-19.27 ng/mL). These concentrations were significantly lower than those found in the remaining groups.

**Figure 2:**
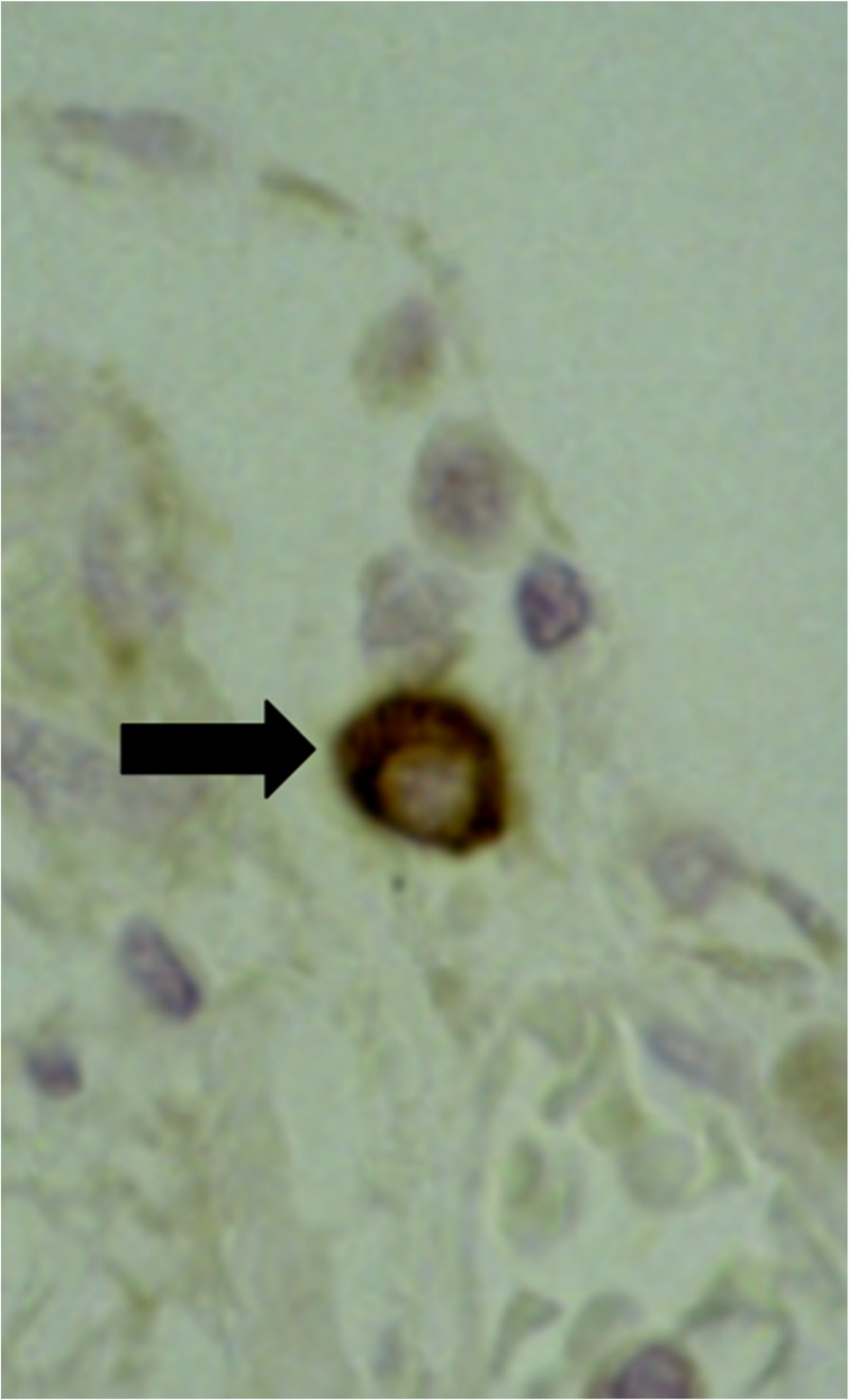
**Spinal cord and meninges of a dog with SRMA.** Arrow indicates TGF-β_1_ positive lymphocyte; immunohistochemistry, anti-TGF-β_1_-ABC, x400. SRMA: steroid-responsive meningitis-arteritis. TGF-β_1_: Transforming growth factor beta 1.

The capture antibody of the commercial human ELISA-Kit succeeded in staining canine TGF-β_1_ in the canine tissues used as positive controls, such as lymph nodes with suppurative lymphadenitis and brain and spinal cord with SRMA (Figure 2). In the negative control sections no TGF-β_1_ was detected.

### Correlation analysis

The data of the parameters correlating with the signalling proteins within the untreated SRMA group are shown in Table 4. A summary of the statistically relevant correlations found in the ‘SRMA’ group is shown in Table 5. A weak positive correlation between signalling protein and IgA concentration was found between CSF TGF-β_1_ and CSF IgA (r_Spear_ = 0.3549; *P* = 0.0337).

**Table 4:** Parameters investigated for the group ‘SRMA’

**Concentration of IgA**		**Number of leukocytes**	
**CSF**	2	**CSF**	1532
(μg/mL)	(0.86-3.1)	cells/3μL)	(548–3720)
**Serum**	167.4	**PB**	25635
(μg/mL)	(98.90-404.5)	(cells/μL)	(17250–31533)

CSF: cerebrospinal fluid; SRMA: steroid-responsive meningitis-arteritis; PB: peripheral blood. Data are given as median and range (25th–75th percentile).

**Table 5:** Significant correlations for the group ‘SRMA’

**Evaluated parameters**		**Spearman’s rank correlation coefficient**
Concentration of IL-6 in CSF	Concentration of VEGF in CSF	0.8246***
Concentration of IL-6 in serum	Concentration of VEGF in serum	0.5045**
Concentration of IL-6 in CSF	Number of leukocytes in CSF	0.8323***
Concentration of VEGF in CSF	Number of leukocytes in CSF	0.5711*
Concentration of TGF-β_1_ in CSF	Concentration of IgA in CSF	0.3549*

SRMA: steroid-responsive meningitis-arteritis; IL-6: interleukin-6; VEGF: vascular endothelial grow factor; TGF-β_1_: Transforming growth factor beta; IgA: immunoglobulin A; CSF: cerebrospinal fluid. Data are given as median and range (25th–75th percentile).

*P < 0.05; ** *P* < 0.01; *** *P* < 0.005.

CSF concentrations of IL-6 and VEGF had a strong positive correlation with the degree of pleocytosis (r_Spear_ = 0.8323; *P* < 0.0001 and r_Spear_ = 0.5711; *P* = 0.0166, respectively). A strong positive correlation was also found between concentration of IL-6 and VEGF, both in CSF (r_Spear_ = 0.8246; *P* < 0,0001) and serum (r_Spear_ = 0.5045; *P =* 0.0086). A similar correlation was not found in serum of ‘Syst Inflam.’ group (r_Spear_ = 0.1340; *P =* 0.339).

## Discussion

The aim of the present study was to investigate the role of IL-6, VEGF and TGF-β_1_ in the pathogenesis of SRMA.

VEGF concentrations were measured, since this parameter has been widely studied in human patients affected with Kawasaki Disease (KD), an acute febrile systemic vasculitis of children. SRMA has been proposed as an animal model for KD [30,31]. In KD patients, concentrations of VEGF were found significantly elevated [16,17,32]. The histopathology of meningeal arteries of dogs euthanised during the acute stage of SRMA typically revealed endothelial and subendothelial oedema, hyaline degeneration and a mild to moderate periarteritis [33]. Therefore, we hypothesized that elevated VEGF might cause these early vascular changes. Indeed, our results showed that VEGF was increased both systemically and intrathecally in SRMA patients during the clinical phases of the disease (‘SRMA’ and ‘SRMA R’). At these time points the mentioned vascular changes can be observed [33].

In other different inflammatory processes within the CNS (“ME” group), VEGF was found to be increased only in single cases, but overall the CSF VEGF concentrations in this group was significantly lower than in the SRMA group. We might conclude that VEGF does not play an important role in other meningoencephalomyelitides. However, due to the heterogeneity of the diseases included in the ME group (e.g. granulomatous meningoencephalomyelitis, necrotising encephalitides and meningoencephalomyelitis of unknown origin) we can not exclude from this study a role for VEGF in some of these diseases.

The values of VEGF in cases with systemic inflammation were lower than in dogs with SRMA (median 3.9 pg/mL and 43.92 pg/mL, respectively). However, the difference was not statistically significant. This is in accordance with a recent study in which VEGF values were elevated in 64% of dogs with systemic inflammatory response syndrome. However, a correlation with clinical signs or increased permeability could not be proved [20].

VEGF is involved in the proposed pathogenesis of vasculitis in KD. After the permeability of the vessels is increased under to the influence of VEGF, platelets might adhere to the vascular wall and inflammatory cells cross the loose endothelium, accumulating in the intima and becoming a source of proinflammatory cytokines. As final consequence there is a thickening of the intima as found in both diseases, KD and SRMA. If coronary artery lesions occur, this might even lead to life-threatening complications [31]. In KD patients VEGF was correlated with the development of coronary artery lesions [32].

Interestingly in our study VEGF was not only found increased in the acute stage of SRMA, but also during relapses of SRMA (median in CSF 20.80 pg/mL; median in serum 33.71 pg/mL). Therefore, VEGF in SRMA may enhance vascular wall destruction in the acute phase of the disease before immune complexes appear [33]. However, further *in vitro* studies evaluating directly the effect of VEGF on canine vasculature (e.g. using endothelial cells culture or cultured vessels) are needed to confirm the role of this protein in the pathogenesis of canine arteritis.

Since VEGF concentrations were increased during relapses, consequently, VEGF may be also involved in the development of the arterial lesions found during the chronic phase, such as increased wall thickness, stenosis and fibrosis [33]. On the other hand, VEGF might indicate simply vascular damage. A limitation of this study is the lack of comparison to other pure vasculitides. Experimental *in vivo* studies are probably necessary to elucidate the long-term effect of VEGF on canine vessels. Experimental studies on SRMA dogs are feasible, due to the natural occurrence and the favourable prognosis of the disease.

The recruitment and activation of different lymphocytes subsets after alteration of the CNS tissue by an environmental factor are caused by multiple mechanisms [34]. These include chemotactic agents [35], probably additional mechanisms such as changes of the blood–brain barrier [36,37], and altered expression of selectins and integrins [38,39]. To add more information to these previous studies and to investigate the hypothesis that IL-6 and TGF-β_1_ are correlated to and eventually involved in the pathogenesis of fever, pleocytosis and increased IgA production in SRMA, these proteins were determined in CSF and serum samples. Previous studies on cytokine expression in SRMA patients, showed an up-regulation of IL-4 and IL-8, while IL-2 and IFN-γ were found in low concentrations [12,35]. Hogenesch et al. [40] investigated IL-6 in serum of dogs with juvenile polyarteritis syndrome and detected increased IL-6 serum values. In preliminary studies, measurement of IL-6 in CSF was considered to be a valuable biomarker for the diagnosis of SRMA [41]. Qualitative studies containing information about the bioactivity of IL-6 in CSF were missing.

In the current study IL-6 values were increased intrathecally and systemically in SRMA patients, the highest concentrations were found in CSF samples (median 1582 pg/mL in ‘SRMA’ and median 637.7 pg/mL in the ‘SRMA R’ groups). In case of other inflammatory diseases of the CNS (‘ME’ group) the concentrations of CSF IL-6 were significantly lower (median 3 pg/mL), leading to the conclusion that IL-6 is an important biomarker for disease activity in SRMA. The exact role of IL-6 in the pathogenesis of the disease could be investigated in experimental studies. Further, IL-6 in SRMA strongly correlated with the degree of pleocytosis. This fact might also suggests, that IL-6 values might be the result of the severe pleocytosis because of its production by macrophages [23]. Lowrie et al. [41] also detected elevated IL-6 CSF values in samples of dogs with a putative relapse and a normal CSF cell count, making the latter hypothesis less likely, nevertheless further studies should be addressed to clarify causes and consequences.

IL-6 has long-range effects, indeed it is one of the most important endogenous pyrogens, induces hepatocytes to synthesize acute-phase proteins, stimulates neutrophil mobilization from bone marrow and stimulates terminal differentiation of B cells to secret immunoglobulins [23,42,43]. Therefore it is very likely that an overproduction of IL-6 is a major mediator of the most peculiar findings, such as fever, increased acute-phase proteins, CSF neutrophilic pleocytosis and peripheral leukocytosis as well as increased IgA production during the acute phase of SRMA. The extreme high values of IL-6 in CSF also during relapses suggest that IL-6 exerts its major functions intrathecally and throughout the waxing and waning course of the disease. As previously mentioned, the cell population in CSF of dogs during the acute phase of SRMA is predominantly composed of neutrophils, during the chronic form macrophages tend to prevail. Upregulation of CD11a on neutrophils [39], increased IL-8 levels in CSF [35] and the destruction of the blood–brain barrier [44] have all been shown to be involved in neutrophil migration into CSF. Factors involved in the accumulation of monocytes in CSF of SRMA patients during the protracted form have not been investigated. Interestingly, IL-6 has been recently proposed to be a regulator of the transition from a neutrophil-dominated to a macrophage-dominated process [45]. We therefore propose a leading role for IL-6 in both the acute and protracted forms of the disease.

As expected, serum IL-6 concentrations were similar to the group of systemic inflammatory diseases supporting other studies, where IL-6 has been used not only as a general marker of inflammation [26], but in particular as a prognostic factor in canine systemic inflammatory response syndrome and sepsis [27]. Also in KD IL-6 is increased in serum, but contrary to VEGF, the increase was not correlated with the development of coronary artery aneurysms and dilatation [46,47].

In SRMA dogs both VEGF and IL-6 were much higher in CSF compared to serum values. This might reflect a more severe inflammatory process affecting meninges and meningeal vessels compared to peripheral vessels, or a main intrathecal production of these signalling proteins, followed by a secondary leakage into the systemic circulation. Further studies including protein associated gene expression and immunohistochemistry of meningeal and peripheral vessels might be necessary to elucidate the site of production.

TGF-β_1_ in SRMA patients was decreased in serum (median 13.57 ng/mL) and elevated in CSF (median 90 pg/mL). The increased concentration in CSF was not specific for SRMA, indeed similar values have been found in other meningoencephalomyelitides (median 58 pg/mL), while the reduced concentration in serum statistically differed from the other groups.

The serum concentrations of TGF-β_1_ were found to be decreased also in patients with KD [48], but to the authors knowledge data concerning concentrations of TGF-β_1_ in CSF of patients with KD are lacking. The hypothesis that TGF-β_1_ might be the most important pathogenetic factor for the excessive IgA production in SRMA could be partially rejected in the current study. Our results support the suggestion that TGF-β_1_ has a minor role in systemic production of IgA, whereas it is highly likely that it might still play a certain role in the intrathecal production of IgA. Indeed, TGF-β_1_ was positively correlated with IgA concentrations in CSF (r_Spear_ = 0.3549; *P* = 0.0337). However, CSF IgA concentrations remain high during teatment [5,8,10] and concentration of TGF-β_1_ decline. Therefore, this rather unspecific elevation of TGF-β_1_ values in CSF samples supports a more immunoregulatory function of this cytokine in inflammatory CNS diseases [49,50]. Further experimental *in vivo* and *in vitro* studies are needed to confirm this hypothesis.

Our findings indicate that the CSF cytokine profile of SRMA dogs during the acute phase is characterized by increased values of IL-6 and TGF-β_1_. Recent progress in immunology led to the discovery of Th17 cells, a new subset of T helper cells [51,52]. According to one study, the combined influence of both IL-6 and TGF-ß_1_ is necessary for the Th17 lineage to develop, while TGF-β_1_ alone shifts the development of naïve T-cells towards T regulatory cells, a T-cell subset that restrains inflammation and maintains tolerance [53].

The detected combined high intrathecal production of TGF-β_1_ and IL-6 in SRMA could possibly lead to an increase of Th17 lymphocyte subset and subsequently enhance the development of an autoimmune response. IL-17, the main product of this lymphocyte subset, plays an active role in inflammatory response and in autoimmune diseases [54] and experimental studies displayed a neutrophil-mobilizing mechanism of IL-17A [55]. The massive invasion of neutrophils into the subarachnoidal space in SRMA dogs might be the results of a Th17 immune response. Further studies to prove direct evidence of Th17 cells and its products in SRMA patients have to be conducted.

To date, SRMA has been believed to be a Th2-mediated immune disorder [12], our results suggest indirectly that in SRMA a Th17 skewed immune response might play a major role, particularly in the development of the meningitis.

## Conclusions

Analysis of the pattern of signalling proteins production in SRMA showed many similarities with results in KD supporting the usefulness of this animal model. In our study increased concentrations of VEGF and IL-6 in serum and CSF of dogs affected with SRMA were found. TGF-β_1_ was increased in CSF and decreased in serum. This study produces evidence that these three signalling proteins are biomarkers of disease activity in SRMA. VEGF might be involved in the pathogenesis of vasculitis, especially in a pronounced permeability and vessel wall damage. TGF-β_1_ is considered to be involved in the excessive IgA production. However, the presented data indicate that additional proteins may influence IgA production. Pleocytosis in SRMA dogs is supported by extremely high intrathecal IL-6 production; a similar pathomechanism might be responsible for the continuous ongoing of the disease and the invasion of neutrophils. The hypothesis that SRMA might be a Th17-mediated disorder should be further investigated.

## Abbreviations

CBC: Complete blood cell count; CNS: Central nervous system; CNS-Mix: Miscellaneous non-inflammatory diseases of the CNS; CSF: Cerebrospinal fluid; ELISA: Enzyme-linked immunosorbent assays; IE: Idiopathic epilepsy; IgA: Immunoglobulin A; IL: Interleukin; KD: Kawasaki Disease; ME: Meningoencephalomyelitides; rcIL-6: Recombinant canine IL-6; r_Spear_: Spearman’s rank correlation coefficient; SRMA: Steroid responsive meningitis-arteritis; SRMA R: SRMA relapse; SRMA Th: SRMA therapy; Syst. Infl.: Systemic inflammatory diseases; TGF-β_1_: Transforming growth factor beta 1; Th: T helper cells; TNF: Tumor necrosis factor; VEGF: Vascular endothelial growth factor.

## Competing interests

The authors declare that they have no competing interests.

## Authors’ contributions

AT designed and supervised the study. AM performed the experiments and analysed the data concerning the part of VEGF and IL-6. OT performed the experiments and analysed the data concerning the part of TGF-β_1_. RC gave substantial contributions to acquisition, analysis and interpretation of the data in all the experiments. MHT provided the laboratory, materials, supervision and substantial contribution to acquisition of the data for the immunohistochemical part of the experiment on TGF-β_1_. AM drafted the manuscript and all authors contributed to the critical revision of the manuscript for important intellectual content and have read and approved the final version.
